# MetaCOXI: an integrated collection of metazoan mitochondrial cytochrome oxidase subunit-I DNA sequences

**DOI:** 10.1093/database/baab084

**Published:** 2022-02-05

**Authors:** Bachir Balech, Anna Sandionigi, Marinella Marzano, Graziano Pesole, Monica Santamaria

**Affiliations:** Institute of Biomembranes, Bioenergetics and Molecular Biotechnologies, National Research Council of Italy, via Amendola 122/O, Bari 70126, Italy; Research and Development Department, Quantia Consulting srl, via Francesco Petrarca 20, Mariano Comense 22066, Italy; Institute of Biomembranes, Bioenergetics and Molecular Biotechnologies, National Research Council of Italy, via Amendola 122/O, Bari 70126, Italy; Institute of Biomembranes, Bioenergetics and Molecular Biotechnologies, National Research Council of Italy, via Amendola 122/O, Bari 70126, Italy; Department of Biosciences, Biotechnology and Biopharmaceutics, University of Bari ‘A. Moro’, via Orabona 4, Bari 70126, Italy; Institute of Biomembranes, Bioenergetics and Molecular Biotechnologies, National Research Council of Italy, via Amendola 122/O, Bari 70126, Italy

## Abstract

Nucleotide sequences reference collections or databases are fundamental components in DNA barcoding and metabarcoding data analyses pipelines. In such analyses, the accurate taxonomic assignment is a crucial aspect, relying directly on the availability of comprehensive and curated reference sequence collection and its taxonomy information. The currently wide use of the mitochondrial cytochrome oxidase subunit-I (COXI) as a standard DNA barcode marker in metazoan biodiversity studies highlights the need to shed light on the availability of the related relevant information from different data sources and their eventual integration. To adequately address data integration process, many aspects should be markedly considered starting from DNA sequence curation followed by taxonomy alignment with solid reference backbone and metadata harmonization according to universal standards. Here, we present MetaCOXI, an integrated collection of curated metazoan COXI DNA sequences with their associated harmonized taxonomy and metadata. This collection was built on the two most extensive available data resources, namely the European Nucleotide Archive (ENA) and the Barcode of Life Data System (BOLD). The current release contains more than 5.6 million entries (39.1% unique to BOLD, 3.6% unique to ENA, and 57.2% shared between both), their related taxonomic classification based on NCBI reference taxonomy, and their available main metadata relevant to environmental DNA studies, such as geographical coordinates, sampling country and host species. MetaCOXI is available in standard universal formats (‘fasta’ for sequences & ‘tsv’ for taxonomy and metadata), which can be easily incorporated in standard or specific DNA barcoding and/or metabarcoding data analysis pipelines.

**Database URL**: https://github.com/bachob5/MetaCOXI

## Introduction

A critical aspect of environmental DNA (eDNA) research is the capacity to collectively characterize the genetic material (intracellular or extracellular) of a variety of living or even dead organisms (e.g. ancient eDNA) in a given sample at taxonomic and functional levels (1–3). The use of such an approach is currently spanned over different scientific disciplines, including biodiversity monitoring programmes, ecosystem services conservation and recovery, environmental health and biomedical research (4, 5). eDNA is the core object of a common DNA metabarcoding experiment aiming at the massive reading of a DNA barcode marker using high-throughput sequencing (HTS) technologies that enables to explore the taxonomic diversity in an environment/habitat of interest (i.e. terrestrial and aquatic) mostly at species level (6, 7). In animals’ DNA barcoding and metabarcoding studies, a fragment, usually the standard Folmer locus, of the cytochrome oxidase subunit-I (COXI) mitochondrial gene is typically sequenced and subsequently assigned to a known taxon (8–12). Indeed, a fundamental requisite to reach confident assignment results is the availability of a comprehensive and curated reference sequence and taxonomy database (12). In this context, recent studies highlight significant gaps of representative sequences for some taxa in DNA databases due to the lack of data integration efforts limiting the study outcomes and their eventual interpretation (13, 14). Enhancing the comprehensiveness of such databases would be among the solutions to fill those gaps in many environmental ecosystem research contexts (e.g. sea water) even at different gradients or habitats (14).

The most used public resources of animals’ COXI sequences are the Barcode of Life Data System (BOLD) (15) and the primary molecular data resources, such as the European Nucleotide Archive (ENA) (16). ENA dump data are open-source, easily retrievable and structured in flat files format. BOLD, instead, contains well-represented and curated sets of COXI sequences, including private subsets. In addition to these two data resources, few are the existing specialized and curated COXI sequences collections, which are essentially based on GenBank (NCBI) data. The first example is Zeale, representing a short fragment (157 bp) of COXI gene sequences belonging to the phylum Arthropoda (17). Midori (18) is another example of mitochondrial genes datasets, including COXI. It is also based only on nucleotide-NCBI (nt) sequences considering mainly the feature table annotations of the entries’ flat files. Besides, database builders, such as CO-Arbitrator (19), were also exploited to generate curated COXI datasets, according to accurate quality parameters, by collecting random sequences from BOLD to query nt-NCBI. Finally, a recently published pipeline, called MARES (20), describes an integration protocol of taxonomy and sequence data from both BOLD and NCBI following a taxonomic quality assessment. However, the procedure focuses only on sequence records belonging to marine ecosystem.

To our knowledge, except MARES, none of the existing resources adopts a sequence data, taxonomy and metadata integration from different data sources with the aim to create a harmonized COXI database or collection. In this perspective, the exponential growth of molecular biodiversity data underlines the urgent need to address taxonomy harmonization issue (i.e. using permanent taxids) across nucleotide reference databases. In addition, harmonization should also include metadata by adopting universal data standards, able to efficiently associate sequence entries to their environmental context (21–24). This would enhance the comprehensiveness and the alignment of such datasets, crucial for DNA barcoding, metabarcoding and eDNA data analyses in general.

In this study, we present MetaCOXI, an integrated collection of Metazoan COXI DNA sequences originated from both ENA and BOLD data entries, generated following an internal data processing workflow, which applies sequence quality assessment, removes entries redundancy, and provides a harmonized taxonomic classification, according to NCBI reference backbone (25) at the main seven levels, with nine associated metadata (mainly relevant to environmental DNA research).

## Materials and methods

### Data sources

To create the present collection, public data entries of BOLD and ENA were exploited. BOLD data (http://www.boldsystems.org/) corresponding to the COXI DNA barcode marker of animals, tagged as ‘COI-5P’, were downloaded (April 2020) in ‘tsv’ format through an Application Programming Interface (API). ENA animals’ divisions (vertebrate, invertebrate, mouse, human and mammals) belonging to the standard release 142 were retrieved (April 2020) in flat files format using ‘wget’ application with file transfer protocol (http://ftp.ebi.ac.uk/pub/databases/ena/sequence/release/std/).

### Data processing

**Figure 1.**
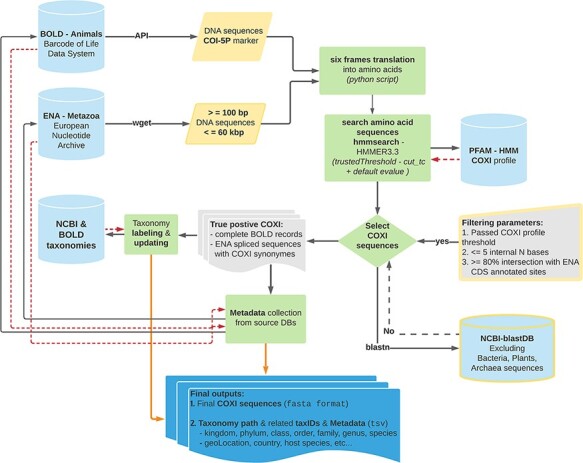
Schematic representation of the bioinformatics workflow used to build MetaCOXI. The whole process was implemented and executed in Linux environment using bash shell commands in combination with custom python scripts to parse the intermediate results. ENA raw sequences were filtered according to their lengths. *hmmsearch* application was used to search the translated DNA sequences against COXI HMM PFAM reference profile. All matches satisfying the TC threshold were selected. The sequences were further validated using blastn to exclude bacterial, plants and Archaea sequences. Taxonomical classification of all entries was aligned to NCBI reference taxonomy. The final COXI sequences and their associated harmonized taxonomy paths and metadata are provided in ‘fasta’ and ‘tsv’ formats, respectively. Yellow color indicates filtering parameters. Green color denotes processing steps. Dashed red arrows illustrate the returning results from reference databases.

BOLD and ENA DNA sequences were analyzed according to the data processing workflow illustrated in Figure 1 (code available at: MetaCOXI_pipeline.sh). In order to exclude very short sequences, nuclear genomes and genomic scaffolds, only sequence lengths ranging from 100 bp to 60 kbp were considered (the latter being the approximate length of the longest mitochondrial genome reported to date (26)). The selected short fragments (less than 500 bp) would potentially benefit the experiments based on DNA mini-barcodes (27, 28) or on reads of typical HTS outputs (29). To avoid the presence of stop codons and to increase the accuracy of matching with the reference COXI profile, all DNA sequences were translated into amino acids, with a custom python script (newTranslator_CExtract.py), using their corresponding mitochondrial genetic code (retrieved from NCBI taxonomy dump files: https://www.ncbi.nlm.nih.gov/taxonomy) and all six open reading frames. The translated sequences were then searched against the reference Hidden Markov Model (HMM) COXI PFAM profile (30) by means of *hmmsearch* application (HMMER3.3 package (31)). All matches that satisfied the COXI HMM profile-specific trusted cutoff (profile internal TC = 34) parameter, also known as ‘*cut_tc*’, were considered true positives. According to HMMER user guide, the TC threshold is generally considered the lowest score known to be true positive (match score above TC threshold in HMM space) that is above all known false positives. Being pre-curated, BOLD entries that passed the TC threshold were fully retained. Analogously, the feature table of ENA true-positive entries flat files was parsed (custom python script: compare_GB_PFAM_annotations_2.py) to get ‘gene’ and ‘product’ features labels and the relative ‘CDS’ (Coding Sequence) positions. Only sequences sharing at least 80% of ENA CDS sites with COXI HMM profile localization were kept and the CDS location was maintained. This comparison allowed also to collect all ‘gene’ or ‘product’ labels that might be considered potential COXI gene name synonyms The selected COXI sequences were further validated, using blastn algorithm (32), to exclude potential matches with bacterial, plants and Archaea sequences of NCBI blast database. Additional sequence rejection criteria regarded the presence of pseudogene tags in the feature table of ENA entries as well as the occurrence of more than five internal ambiguous nucleotide (‘N’) bases after terminal ‘Ns’ trimming.

### Data integration and harmonization

To create the integrated collection, the final selected entries of both data sources were combined and subsequently dereplicated according to their related accession numbers. BOLD records without accession numbers were labeled with their provided process ID. Taxonomy path labeling was harmonized for all entries according to NCBI taxonomy reference by retrieving the taxonomy id (taxid) associated with the ENA accession number (https://ftp.ncbi.nih.gov/pub/taxonomy/accession2taxid/) and its corresponding taxonomic classification at the main seven levels: kingdom, phylum, class, order, family, genus and species. A necessary update was performed on some BOLD entries where according to NCBI backbone a different taxonomy path, most probably due to recent names change, was still reported. Furthermore, BOLD entries lacking associated ENA accession numbers (BOLD’s exclusive entries) were updated by reporting the classification of the lowest rank taxon name present in their taxonomy path (i.e. species, genus, order, etc.). Finally, taxon names not found in the reference taxonomy were kept unchanged and their taxids were labeled as ‘NA’. Many reasons are behind missing taxonomic names in NCBI taxonomy such as a recent update of the taxon lineage or the absence of the specific rank’s name (e.g. unknown or unreported phylum name for a given species). In addition to the taxonomy, the available associated metadata were collected from ENA flat files feature table and from the raw BOLD data files. These consisted of (i) the final sequence length, (ii) the mitochondrial genetic code, (iii) the sampling country name, (iv) sampling country address, (v) host species, (vi) collection year, (vii) collection complete date, (viii) geographical coordinates (harmonized in decimal degrees—DD format) and (ix) the web permanent link to the source database entry.

## Results

The current release of MetaCOXI includes 5 608 848 curated COXI sequences freely available in ‘fasta’ format (‘MetaCOXI_Seqs.tar.gz’). As shown in Table 1, approximately 57% of the entries originated from both databases, while 39% were unique to BOLD and 3.6% to ENA. Out of 5 421 628 retrieved BOLD entries only 7432 did not satisfy the TC threshold. This indicates that the approach of using the COXI profile-specific cutoff threshold had an additional conservative effect, which increased the accuracy of determining a true positive match by 0.14%. Apart from those not satisfying the applied TC threshold, additional investigation revealed that some of the rejected BOLD entries (916) presented one or more internal stop codons in their amino acid sequences. Such assessment was not possible to conduct on ENA sequences as their identity of coding for COXI gene was inferred through the present analyses. Importantly, according to the feature tables of selected ENA entries, a total of 160 potential COXI ‘gene’ or ‘product’ names synonyms were collected (Supplementary Table S1). These would be useful not only for future automation of COXI data retrieval but also to maximize the returning results of real available data of this gene when using the public API of ENA and/or NCBI primary databases.

**Table 1.** Number of entries in MetaCOXI related to their source DB: BOLD, ENA or both

MetaCOXI	BOLD unique	ENA unique	BOLD and ENA
5 608 848	2 195 176 (39.13%)	201 719 (3.59%)	3 211 953 (57.26%)

**Figure 2.**
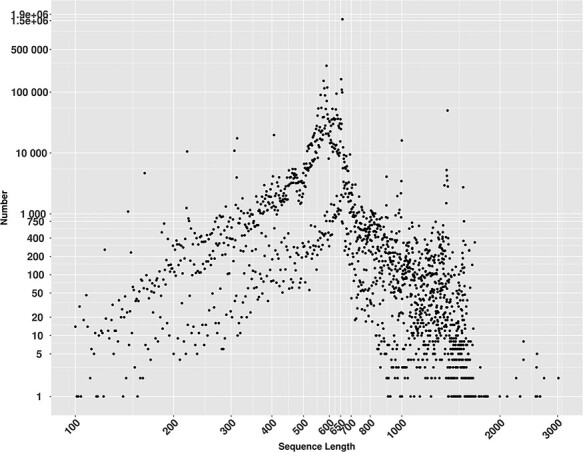
Lengths distribution of MetaCOXI sequences ranging from 100 to 3020 bp. The most frequent sequence length is 658 bp represented by 1 573 982 sequences.

MetaCOXI sequences length distribution ranged from 100 to 3020 bp (Figure 2), where 658 bp, the known length of the standard DNA barcode region in animals (8), is the most frequent and represented by more than 1.5 million (1 573 982) sequences.

Regarding taxonomy, the collection contains sequences of 743 716 scientific species names, which were labeled following the alignment of NCBI and BOLD taxonomies. These correspond to 52 250 genera, 5123 families, 617 orders, 100 classes and 31 phyla. All taxa names and their taxids are provided in ‘tsv’ format file (‘MetaCOXI_Taxonomy_Metadata.tar.gz’). In terms of species number, the most represented phyla (Table 2) are Arthropoda (88.6%) followed by Chordata (5.36%), Mollusca (2.56%) and Annelida (1.10%). Insecta and Arachnida are the most represented classes for Arthropods by 76.6% and 6.9%, respectively, while Actinopteri (3.14%) included the highest species number in Chordata, Gastropoda (2.01%) in Mollusca and Polychaeta (0.57%) in Annelida (additional details for all classes are available in Supplementary Table S2).

**Table 2.** Taxonomic composition of MetaCOXI at the main six taxonomy levels belonging to Metazoa kingdom with their corresponding sequence number. The percentages are relative to the totals present in the whole collection. Phylum with more than three classes are highlighted in red

Phylum	Seq N°	Class N°	Class %	Order N°	Order %	Family N°	Family %	Genus N°	Genus %	Species N°	Species %
Arthropoda	4 782 392	19	19	130	21.07	2306	45.013	36 559	69.969	658 938	88.601
Chordata	482 868	18	18	171	27.71	1051	20.515	7895	15.110	39 910	5.366
Mollusca	170 563	8	8	80	12.97	587	11.458	3471	6.643	19 049	2.561
Platyhelminthes	28 693	6	6	39	6.32	191	3.728	620	1.187	3228	0.434
Cnidaria	17 787	6	6	24	3.89	229	4.470	749	1.433	3919	0.527
Echinodermata	27 995	5	5	42	6.81	143	2.791	616	1.179	2330	0.313
Porifera	4150	4	4	29	4.70	100	1.952	330	0.632	1465	0.197
Acanthocephala	1944	4	4	7	1.13	18	0.351	54	0.103	162	0.022
Brachiopoda	167	3	3	5	0.81	15	0.293	26	0.050	77	0.010
Bryozoa	2224	3	3	4	0.65	61	1.191	110	0.211	282	0.038
Rotifera	8971	3	3	6	0.97	24	0.468	67	0.128	995	0.134
Nemertea	3849	3	3	4	0.65	32	0.625	88	0.168	703	0.095
Annelida	49 906	3	3	30	4.86	134	2.616	909	1.740	8224	1.106
Hemichordata	131	2	2	2	0.32	5	0.098	8	0.015	30	0.004
Ctenophora	200	2	2	5	0.81	5	0.098	6	0.011	26	0.003
Nematoda	20 979	2	2	19	3.08	148	2.889	499	0.955	3247	0.437
Tardigrada	1705	2	2	5	0.81	8	0.156	42	0.080	319	0.043
Kinorhyncha	390	2	2	0	0.00	8	0.156	8	0.015	36	0.005
Priapulida	17	1	1	1	0.16	2	0.039	4	0.008	4	0.001
Onychophora	1235	1	1	1	0.16	2	0.039	33	0.063	199	0.027
Chaetognatha	1098	1	1	3	0.49	6	0.117	15	0.029	97	0.013
Nematomorpha	328	1	1	2	0.32	3	0.059	8	0.015	87	0.012
Entoprocta	33	0	0	0	0.00	3	0.059	7	0.013	23	0.003
Gastrotricha	451	0	0	2	0.32	11	0.215	33	0.063	124	0.017
Sipuncula	4	0	0	0	0.00	0	0.000	0	0.000	0	0.000
Placozoa	11	0	0	0	0.00	1	0.020	1	0.002	6	0.001
Dicyemida	102	0	0	0	0.00	1	0.020	2	0.004	16	0.002
Gnathostomulida	14	0	0	2	0.32	6	0.117	8	0.015	10	0.001
Xenacoelomorpha	261	0	0	2	0.32	20	0.390	48	0.092	175	0.024
Cycliophora	267	0	0	0	0.00	0	0.000	1	0.002	5	0.001
Phoronida	106	0	0	0	0.00	0	0.000	2	0.004	23	0.003
NA	7	1	1	1	0.16	1	0.020	1	0.002	7	0.001

Taxonomy harmonization implied the update of more than 1.9 million (1,958,146) BOLD records where at least one taxon name has been changed/updated when compared to NCBI taxonomy (refer to the file ‘UpdatedBOLD_Taxonomy.tsv.tar.gz’). As for metadata, the sampling ‘country’ name was found for 5 031 581 records, geographical coordinates for 4 348 624 and host species for 146 906 (an example of metadata file is available at: ‘Sample_Metadata.tsv’).

## Conclusion and future directions

As molecular biodiversity studies are increasingly gaining attentions, their associated data are growing at considerable levels. The present study describes and provides a valuable COXI DNA sequences-integrated collection resource, MetaCOXI, characterized by harmonized taxonomy and metadata. Upon building this collection, several fundamental aspects and urgent initiatives were highlighted, including the need to use harmonized and standardized taxonomy and metadata, such as adopting permanent, aligned and global taxids to represent taxonomic classifications. As MetaCOXI is delivered in standard formats (‘fasta’ for sequences and ‘tsv’ for taxonomy and metadata), it can be easily adapted to any DNA barcoding or metabarcoding experiment involving the mitochondrial COXI gene. For instance, the end user can easily extract the sequences of specific lengths (e.g. longer than 500 bp) or the data belonging to the lineages of his interest (e.g. specific natural ecosystem). In this context, the collection can be readily exploited in standard or specific data analysis pipelines as it is compatible with the data types requested by several algorithms used in DNA barcoding and metabarcoding such as Usearch or Uclust (33), Blast (32) or others. Although MetaCOXI respects all FAIR (24) data principles being easily findable, freely accessible, interoperable and reusable, these aspects will be further improved and complemented by the development of a user-friendly web interface and an API, which are planned for the next releases. Moreover, the core pipeline of MetaCOXI is in continuous growth and optimization where further implementations will include the release of a dereplicated version with additional standard formats as required by many DNA metabarcoding data analysis pipelines. The collection will be updated at 6 months-based intervals following the availability of new ENA standard releases.

## Supplementary Material

baab084_Supp

## References

[1] Taberlet P. , Coissac E., Pompanon F. et al. (2012) Towards next-generation biodiversity assessment using DNA metabarcoding. *Mol. Ecol.*, 21, 2045–2050. 22486824 10.1111/j.1365-294X.2012.05470.x

[2] Seymour M. , Edwards F.K., Cosby B.J. et al. (2021) Environmental DNA provides higher resolution assessment of riverine biodiversity and ecosystem function via spatio-temporal nestedness and turnover partitioning. *Commun. Biol.*, 4, 512. 10.1038/s42003-021-02031-2 PMC8093236 33941836

[3] Pawlowski J. , Apothéloz-Perret-Gentil L. and Altermatt F. (2020) Environmental DNA: what’s behind the term? Clarifying the terminology and recommendations for its future use in biomonitoring. *Mol. Ecol.*, 29, 4258–4264. 32966665 10.1111/mec.15643

[4] Willis J.R. and Gabaldón T. (2020) The human oral microbiome in health and disease: from sequences to ecosystems. *Microorganisms*, 8, 308. 10.3390/microorganisms8020308 PMC7074908 32102216

[5] Ruppert K.M. , Kline R.J. and Rahman M.S. (2019) Past, present, and future perspectives of environmental DNA (eDNA) metabarcoding: a systematic review in methods, monitoring, and applications of global eDNA. *Glob. Ecol. Conserv.*, 17, e00547.

[6] Zinger L. , Bonin A., Alsos I.G. et al. (2019) DNA metabarcoding—Need for robust experimental designs to draw sound ecological conclusions. *Mol. Ecol.*, 28, 1857–1862. 31033079 10.1111/mec.15060

[7] Deiner K. , Bik H.M., Mächler E. et al. (2017) Environmental DNA metabarcoding: transforming how we survey animal and plant communities. *Mol. Ecol.*, 26, 5872–5895. 28921802 10.1111/mec.14350

[8] Hebert P.D.N. , Cywinska A., Ball S.L. et al. (2003) Biological identifications through DNA barcodes. *Proc. R. Soc. B Biol. Sci.*, 270, 313–321. 10.1098/rspb.2002.2218 PMC1691236 12614582

[9] Balech B. , Sandionigi A., Manzari C. et al. (2018) Tackling critical parameters in metazoan meta-barcoding experiments: a preliminary study based on coxI DNA barcode. *PeerJ*, 6, e4845. 10.7717/peerj.4845 PMC6004112 29915686

[10] Dopheide C. , Tooman L., Grosser S. et al. (2019) Estimating the biodiversity of terrestrial invertebrates on a forested Island using DNA barcodes and metabarcoding data. *Ecol. Appl.*, 29, e01877, 1–14. 10.1002/eap.1877 30811075

[11] Tizard J. , Patel S., Waugh J. et al. (2019) DNA barcoding a unique avifauna: an important tool for evolution, systematics and conservation. *BMC Evol. Biol.*, 19, 1–13. 10.1186/s12862-019-1346-y PMC6369544 30744573

[12] Hajibabaei M. (2012) The golden age of DNA metasystematics. *Trends Genet.*, 28, 535–537. 22951138 10.1016/j.tig.2012.08.001

[13] Hestetun J.T. , Bye-Ingebrigtsen E., Nilsson R.H. et al. (2020) Significant taxon sampling gaps in DNA databases limit the operational use of marine macrofauna metabarcoding. *Mar. Biodivers.*, 50, 1–9.

[14] Wangensteen O. , Palacín C., Guardiola M. et al. (2018) DNA metabarcoding of littoral hard-bottom communities: high diversity and database gaps revealed by two molecular markers. *PeerJ*, 6, e4705. 10.7717/peerj.4705 PMC5937484 29740514

[15] Ratnasingham S. and Hebert P.D.N. (2007) BOLD: the barcode of life data system: barcoding. *Mol. Ecol. Notes*, 7, 355–364. 18784790 10.1111/j.1471-8286.2007.01678.x PMC1890991

[16] Leinonen R. , Akhtar R., Birney E. et al. (2011) The European nucleotide archive. *Nucleic Acids Res.*, 39, D28. 10.1093/nar/gkq967 PMC3013801 20972220

[17] Richardson R.T. , Bengtsson-Palme J., Gardiner M.M. et al. (2018) A reference cytochrome c oxidase subunit I database curated for hierarchical classification of arthropod metabarcoding data. *PeerJ*, 2018, e5126. 10.7717/peerj.5126 PMC6025149 29967752

[18] Machida R.J. , Leray M., Ho S.-L. et al. (2017) Metazoan mitochondrial gene sequence reference datasets for taxonomic assignment of environmental samples. *Sci. Data*, 4, 170027. 10.1038/sdata.2017.27 PMC5349245 28291235

[19] Heller P. , Casaletto J., Ruiz G. et al. (2018) A database of metazoan cytochrome c oxidase subunit I gene sequences derived from GenBank with CO-ARBitrator. *Sci. Data*, 5, 1–7. 30084847 10.1038/sdata.2018.156 PMC6080493

[20] Arranz V. , Pearman W.S., Aguirre J.D. et al. (2020) MARES, a replicable pipeline and curated reference database for marine eukaryote metabarcoding. *Sci. Data*, 7, 1–8. 32620910 10.1038/s41597-020-0549-9 PMC7334202

[21] Damerow J.E. , Varadharajan C., Boye K. et al. (2021) Sample identifiers and metadata to support data management and reuse in multidisciplinary ecosystem sciences. *Data Sci. J.*, 20, 1–19.

[22] Yilmaz P. , Kottmann R., Field D. et al. (2011) Minimum information about a marker gene sequence (MIMARKS) and minimum information about any (x) sequence (MIxS) specifications. *Nat. Biotechnol.*, 29, 415–420. 21552244 10.1038/nbt.1823 PMC3367316

[23] Buttigieg P.L. , Pafilis E., Lewis S.E. et al. (2016) The environment ontology in 2016: bridging domains with increased scope, semantic density, and interoperation. *J. Biomed. Semantics*, 7, 1–12. 10.1186/s13326-016-0097-6 PMC5035502 27664130

[24] Wilkinson M.D. , Dumontier M., Aalbersberg I.J. et al. (2016) Comment: the FAIR Guiding Principles for scientific data management and stewardship. *Sci. Data*, 3, 1–9. 10.1038/sdata.2016.18 PMC4792175 26978244

[25] Schoch C.L. , Ciufo S., Domrachev M. et al. (2020) NCBI Taxonomy: a comprehensive update on curation, resources and tools. *Database*, 2020, baaa062, 1–21. 10.1093/database/baaa062 PMC7408187 32761142

[26] Kong L. , Li Y., Kocot K.M. et al. (2020) Mitogenomics reveals phylogenetic relationships of Arcoida (Mollusca, Bivalvia) and multiple independent expansions and contractions in mitochondrial genome size. *Mol. Phylogenet. Evol.*, 150, 106857. 10.1016/j.ympev.2020.106857 32473333

[27] Shokralla S. , Hellberg R., Handy S. et al. (2015) A DNA mini-barcoding system for authentication of processed fish products. *Sci. Rep.*, 5, 15894, 1–11. 10.1038/srep15894 PMC4626862 26516098

[28] Hajibabaei M. and McKenna C. (2012) DNA mini-barcodes. *Methods Mol. Biol.*, 858, 339–353. 22684963 10.1007/978-1-61779-591-6_15

[29] Palumbo F. , Scariolo F., Vannozzi A. et al. (2020) NGS-based barcoding with mini- COI gene target is useful for pet food market surveys aimed at mislabelling detection. *Sci. Rep.*, 10, 1–8. 33082418 10.1038/s41598-020-74918-9 PMC7575603

[30] Mistry J. , Chuguransky S., Williams L. et al. (2021) Pfam: the protein families database in 2021. *Nucleic Acids Res.*, 49, D412–D419. 33125078 10.1093/nar/gkaa913 PMC7779014

[31] Eddy S.R. (2011) Accelerated profile HMM searches. *PLoS Comput. Biol.*, 7, 1002195. 10.1371/journal.pcbi.1002195 PMC3197634 22039361

[32] Camacho C. , Coulouris G., Avagyan V. et al. (2009) BLAST+: architecture and applications. *BMC Bioinform.*, 10, 421. 10.1186/1471-2105-10-421 PMC2803857 20003500

[33] Edgar R.C. (2010) Search and clustering orders of magnitude faster than BLAST. *Bioinformatics*, 26, 2460–2461. 20709691 10.1093/bioinformatics/btq461

