# Supplementary material for: MetaCOXI: an integrated collection of metazoan mitochondrial cytochrome oxidase subunit-I DNA sequences
Source: Database (Oxford). 2022 Feb 5;2022:baab084. doi: 10.1093/database/baab084 (PMC9216479; doi:10.1093/database/baab084)
Supplement: baab084_Supp [file baab084_supp.zip › baab084_Supp/Supplementary table 1.docx]

**Supplementary table 1.** Gene and product labels recovered from ENA feature tables corresponding to the final MetaCOXI sequences which positively passed the quality filtering process. In total, 160 labels were claimed: 90 ‘gene’ and 70 ‘product’.

| **GENE** |  |  | **PRODUCT** |  |
| --- | --- | --- | --- | --- |
| 1. -CO1 | 46. Ddmco |  | 1. CO1 | 36. cytochrome c oxydase subunit I |
| 2. 1 | 47. Dnpi\mt:CoI |  | 2. COI | 37. cytochrome coxidase subunit I |
| 3. AcCOI | 48. Dsil\mt:CoI |  | 3. COI protein | 38. cytochrome oxidase |
| 4. ApCOI | 49. DtCOI |  | 4. Cytochrome C oxidase subunit 1 (COXI) | 39. cytochrome oxidase 1 |
| 5. AyCOI | 50. Fcox1 |  | 5. Cytochrome c oxidase | 40. cytochrome oxidase 1 subunit |
| 6. BmaCOI | 51. HcCOI |  | 6. Cytochrome c oxidase subunit 1 | 41. cytochrome oxidase I |
| 7. BmoCOI | 52. LP12.1 |  | 7. Cytochrome c oxidase subunit I | 42. cytochrome oxidase I subunit |
| 8. C01 | 53. MT-CO1 |  | 8. Cytochrome oxidase c subunit I | 43. cytochrome oxidase I subunit I |
| 9. C0I | 54. Mcox1 |  | 9. Cytochrome oxidase subunit I | 44. cytochrome oxidase b subunit I |
| 10. C0X1 | 55. PmCOX1 |  | 10. HMEL001510-PA | 45. cytochrome oxidase c |
| 11. CO 1 | 56. RfCOI |  | 11. co1 | 46. cytochrome oxidase c subunit 1 |
| 12. CO I | 57. ScpCOI |  | 12. cox1 | 47. cytochrome oxidase c subunit I |
| 13. CO-1 | 58. ScrCOI |  | 13. cyctochrome c oxidase subunit I | 48. cytochrome oxidase sububit I |
| 14. CO-I | 59. c |  | 14. cytchrome oxidase subunit I | 49. cytochrome oxidase subunit 1 |
| 15. CO1 | 60. c01 |  | 15. cythochrome oxidase subunit I | 50. cytochrome oxidase subunit 1 (COI) |
| 16. CO1, COX1 | 61. cOXI |  | 16. cytochome oxidase subunit I | 51. cytochrome oxidase subunit I |
| 17. CO1X | 62. co 1 |  | 17. cytochrom oxidase I | 52. cytochrome oxidase subunit I (CO1) gene |
| 18. CO2 | 63. co I |  | 18. cytochrome C oxidase | 53. cytochrome oxidase subunit I (COI) |
| 19. COI | 64. co1 |  | 19. cytochrome C oxidase subunit 1 | 54. cytochrome oxidase subunit I-1 |
| 20. COI 1 | 65. coI |  | 20. cytochrome C oxidase subunit I | 55. cytochrome oxidase subunit I-2 |
| 21. COI-LCO | 66. coi |  | 21. cytochrome C oxidase subunit I-like protein | 56. cytochrome oxidase subunit I-like protein |
| 22. COI-PAT | 67. cox |  | 22. cytochrome c oxdiase subunit I | 57. cytochrome oxidase subunit i |
| 23. COI1 | 68. cox 1 |  | 23. cytochrome c oxidas subunit 1 | 58. cytochrome oxidase subunit one |
| 24. COIl | 69. cox I |  | 24. cytochrome c oxidasa subunit I | 59. cytochrome oxidase subunit-1 |
| 25. COX | 70. cox-1 |  | 25. cytochrome c oxidase | 60. cytochrome oxidase, subunit 1 |
| 26. COX 1 | 71. cox1 |  | 26. cytochrome c oxidase 1 | 61. cytochrome oxydase subunit 1 |
| 27. COX I | 72. cox1b |  | 27. cytochrome c oxidase I | 62. cytochrome-c oxidase I |
| 28. COX-1 | 73. coxA |  | 28. cytochrome c oxidase polypeptide | 63. cytochrome-c oxidase chain I |
| 29. COX-I | 74. coxI |  | 29. cytochrome c oxidase polypeptide I | 64. cytochrome-c oxidase subunit I |
| 30. COX1 | 75. coxi |  | 30. cytochrome c oxidase subunit | 65. cytochrone oxidase subunit I |
| 31. COXI | 76. cytb |  | 31. cytochrome c oxidase subunit 1 | 66. cytocrome oxidase subunit I |
| 32. COl | 77. cytc |  | 32. cytochrome c oxidase subunit I | 67. cytohrome oxidase subunit 1 |
| 33. CYCS | 78. cytochrome c oxidase subunit I, COI |  | 33. cytochrome c oxidase subunit I (COI) | 68. cytomchrome c oxidase subunit 1 |
| 34. CjCOI | 79. cytochrome oxidase subunit 1 |  | 34. cytochrome c oxidase subunit I-like protein | 69. cytrochrome oxidase subunit I |
| 35. Co I | 80. cytochrome oxidase subunit I |  | 35. cytochrome c oxidase subunite 1 | 70. cytochrome c oxidasese subunit I |
| 36. Co1 | 81. cytochrome oxidase subunit I (COI) |  |  |  |
| 37. CoI | 82. genomic DNA |  |  |  |
| 38. Coi | 83. mt COI |  |  |  |
| 39. Cox 1 | 84. mt-Co1 |  |  |  |
| 40. Cox I | 85. mt-co1 |  |  |  |
| 41. Cox-1 | 86. mtCO1 |  |  |  |
| 42. Cox1 | 87. mtCOI |  |  |  |
| 43. Cox2 | 88. mtCOX1 |  |  |  |
| 44. CoxI | 89. rag1 |  |  |  |
| 45. Dcyr\mt:CoI | 90. Cytb |  |  |  |
