# Supplementary material for: MetaCOXI: an integrated collection of metazoan mitochondrial cytochrome oxidase subunit-I DNA sequences
Source: Database (Oxford). 2022 Feb 5;2022:baab084. doi: 10.1093/database/baab084 (PMC9216479; doi:10.1093/database/baab084)
Supplement: baab084_Supp [file baab084_supp.zip › baab084_Supp/Supplementary table 2.docx]

**Supplementary table 2.** Phylum and class levels taxonomic composition of MetaCOXI and their relative number of descendent taxonomic ranks. The percentages are relative to the totals present in the whole collection.

| **Phylum** | **Class** | **Orders N°** | **Orders %** | **Families N°** | **Families %** | **Genera N°** | **Genera %** | **Species N°** | **Species %** |
| --- | --- | --- | --- | --- | --- | --- | --- | --- | --- |
| **Arthropoda** | **Insecta** | 30 | 4.862 | 1067 | 20.828 | 30366 | 58.117 | **570403** | **76.6963** |
|  | Hexanauplia | 20 | 3.241 | 167 | 3.260 | 532 | 1.018 | 2807 | 0.3774 |
|  | **Arachnida** | 16 | 2.593 | 518 | 10.111 | 2974 | 5.692 | **51643** | **6.9439** |
|  | **Malacostraca** | 16 | 2.593 | 379 | 7.398 | 1969 | 3.768 | **14190** | **1.9080** |
|  | Diplopoda | 13 | 2.107 | 49 | 0.956 | 187 | 0.358 | 932 | 0.1253 |
|  | Branchiopoda | 7 | 1.135 | 26 | 0.508 | 95 | 0.182 | 1690 | 0.2272 |
|  | Chilopoda | 5 | 0.810 | 18 | 0.351 | 76 | 0.145 | 664 | 0.0893 |
|  | **Collembola** | 4 | 0.648 | 23 | 0.449 | 147 | 0.281 | **15247** | **2.0501** |
|  | Ichthyostraca | 4 | 0.648 | 8 | 0.156 | 11 | 0.021 | 46 | 0.0062 |
|  | Ostracoda | 3 | 0.486 | 21 | 0.410 | 113 | 0.216 | 618 | 0.0831 |
|  | Protura | 2 | 0.324 | 6 | 0.117 | 24 | 0.046 | 61 | 0.0082 |
|  | Diplura | 2 | 0.324 | 0 | 0.000 | 0 | 0.000 | 0 | 0.0000 |
|  | Pycnogonida | 1 | 0.162 | 11 | 0.215 | 35 | 0.067 | 350 | 0.0471 |
|  | NA | 1 | 0.162 | 5 | 0.098 | 10 | 0.019 | 221 | 0.0297 |
|  | Remipedia | 1 | 0.162 | 3 | 0.059 | 8 | 0.015 | 25 | 0.0034 |
|  | Pauropoda | 1 | 0.162 | 2 | 0.039 | 5 | 0.010 | 17 | 0.0023 |
|  | Symphyla | 1 | 0.162 | 2 | 0.039 | 3 | 0.006 | 16 | 0.0022 |
|  | Merostomata | 1 | 0.162 | 1 | 0.020 | 3 | 0.006 | 4 | 0.0005 |
|  | Cephalocarida | 1 | 0.162 | 1 | 0.020 | 2 | 0.004 | 3 | 0.0004 |
|  | Mystacocarida | 1 | 0.162 | 1 | 0.020 | 1 | 0.002 | 1 | 0.0001 |
| **Chordata** | **Actinopteri** | 69 | 11.183 | 490 | 9.565 | 3905 | 7.474 | **23409** | **3.1476** |
|  | **Aves** | 38 | 6.159 | 186 | 3.631 | 1707 | 3.267 | **5428** | **0.7298** |
|  | **Mammalia** | 26 | 4.214 | 150 | 2.928 | 947 | 1.812 | **3323** | **0.4468** |
|  | Chondrichthyes | 13 | 2.107 | 56 | 1.093 | 185 | 0.354 | 1212 | 0.1630 |
|  | Actinopterygii | 5 | 0.810 | 0 | 0.000 | 0 | 0.000 | 0 | 0.0000 |
|  | Amphibia | 3 | 0.486 | 69 | 1.347 | 436 | 0.834 | 3260 | 0.4383 |
|  | Ascidiacea | 3 | 0.486 | 17 | 0.332 | 62 | 0.119 | 424 | 0.0570 |
|  | Lepidosauria | 2 | 0.324 | 54 | 1.054 | 522 | 0.999 | 2340 | 0.3146 |
|  | NA | 2 | 0.324 | 17 | 0.332 | 102 | 0.195 | 378 | 0.0508 |
|  | Dipnoi | 2 | 0.324 | 3 | 0.059 | 3 | 0.006 | 8 | 0.0011 |
|  | Thaliacea | 2 | 0.324 | 2 | 0.039 | 4 | 0.008 | 8 | 0.0011 |
|  | Hyperoartia | 1 | 0.162 | 2 | 0.039 | 9 | 0.017 | 52 | 0.0070 |
|  | Myxini | 1 | 0.162 | 1 | 0.020 | 5 | 0.010 | 36 | 0.0048 |
|  | Leptocardii | 1 | 0.162 | 1 | 0.020 | 3 | 0.006 | 11 | 0.0015 |
|  | Cladistia | 1 | 0.162 | 1 | 0.020 | 2 | 0.004 | 14 | 0.0019 |
|  | Appendicularia | 1 | 0.162 | 1 | 0.020 | 2 | 0.004 | 5 | 0.0007 |
|  | Coelacanthimorpha | 1 | 0.162 | 1 | 0.020 | 1 | 0.002 | 2 | 0.0003 |
|  | Reptilia | 0 | 0.000 | 0 | 0.000 | 0 | 0.000 | 0 | 0.0000 |
|  | Elasmobranchii | 0 | 0.000 | 0 | 0.000 | 0 | 0.000 | 0 | 0.0000 |
| **Mollusca** | **Gastropoda** | 32 | 5.186 | 409 | 7.984 | 2665 | 5.100 | **14962** | **2.0118** |
|  | **Bivalvia** | 29 | 4.700 | 96 | 1.874 | 582 | 1.114 | **3005** | **0.4041** |
|  | **Cephalopoda** | 10 | 1.621 | 45 | 0.878 | 149 | 0.285 | **775** | **0.1042** |
|  | Solenogastres | 3 | 0.486 | 10 | 0.195 | 9 | 0.017 | 14 | 0.0019 |
|  | Polyplacophora | 2 | 0.324 | 16 | 0.312 | 46 | 0.088 | 239 | 0.0321 |
|  | Scaphopoda | 2 | 0.324 | 6 | 0.117 | 11 | 0.021 | 24 | 0.0032 |
|  | Caudofoveata | 2 | 0.324 | 4 | 0.078 | 6 | 0.011 | 22 | 0.0030 |
|  | Monoplacophora | 1 | 0.162 | 1 | 0.020 | 3 | 0.006 | 5 | 0.0007 |
|  | NA | 0 | 0.000 | 0 | 0.000 | 0 | 0.000 | 3 | 0.0004 |
| **Platyhelminthes** | **Cestoda** | 19 | 3.079 | 45 | 0.878 | 192 | 0.367 | **641** | **0.0862** |
|  | **Monogenea** | 8 | 1.297 | 28 | 0.547 | 84 | 0.161 | **608** | **0.0818** |
|  | Rhabditophora | 7 | 1.135 | 46 | 0.898 | 113 | 0.216 | 582 | 0.0783 |
|  | **Trematoda** | 5 | 0.810 | 70 | 1.366 | 228 | 0.436 | **1372** | **0.1845** |
|  | Catenulida | 0 | 0.000 | 2 | 0.039 | 3 | 0.006 | 16 | 0.0022 |
|  | NA | 0 | 0.000 | 0 | 0.000 | 0 | 0.000 | 7 | 0.0009 |
|  | Turbellaria | 0 | 0.000 | 0 | 0.000 | 0 | 0.000 | 2 | 0.0003 |
| **Cnidaria** | **Anthozoa** | 9 | 1.459 | 117 | 2.284 | 490 | 0.938 | **2739** | **0.3683** |
|  | **Hydrozoa** | 6 | 0.972 | 82 | 1.601 | 212 | 0.406 | **848** | **0.1140** |
|  | Scyphozoa | 3 | 0.486 | 16 | 0.312 | 29 | 0.056 | 267 | 0.0359 |
|  | Myxozoa | 3 | 0.486 | 3 | 0.059 | 4 | 0.008 | 13 | 0.0017 |
|  | Cubozoa | 2 | 0.324 | 6 | 0.117 | 7 | 0.013 | 17 | 0.0023 |
|  | Staurozoa | 1 | 0.162 | 5 | 0.098 | 7 | 0.013 | 32 | 0.0043 |
|  | NA | 0 | 0.000 | 0 | 0.000 | 0 | 0.000 | 3 | 0.0004 |
| Echinodermata | Echinoidea | 17 | 2.755 | 41 | 0.800 | 113 | 0.216 | 373 | 0.0502 |
|  | Asteroidea | 8 | 1.297 | 29 | 0.566 | 144 | 0.276 | 510 | 0.0686 |
|  | Ophiuroidea | 6 | 0.972 | 22 | 0.429 | 158 | 0.302 | 829 | 0.1115 |
|  | Holothuroidea | 6 | 0.972 | 22 | 0.429 | 101 | 0.193 | 408 | 0.0549 |
|  | Crinoidea | 5 | 0.810 | 29 | 0.566 | 100 | 0.191 | 201 | 0.0270 |
|  | NA | 0 | 0.000 | 0 | 0.000 | 0 | 0.000 | 9 | 0.0012 |
| **Porifera** | **Demospongiae** | 21 | 3.404 | 81 | 1.581 | 271 | 0.519 | **1333** | **0.1792** |
|  | Hexactinellida | 4 | 0.648 | 14 | 0.273 | 49 | 0.094 | 71 | 0.0095 |
|  | Calcarea | 3 | 0.486 | 3 | 0.059 | 3 | 0.006 | 3 | 0.0004 |
|  | Homoscleromorpha | 1 | 0.162 | 2 | 0.039 | 7 | 0.013 | 55 | 0.0074 |
|  | NA | 0 | 0.000 | 0 | 0.000 | 0 | 0.000 | 3 | 0.0004 |
| Acanthocephala | Archiacanthocephala | 3 | 0.486 | 3 | 0.059 | 6 | 0.011 | 14 | 0.0019 |
|  | Palaeacanthocephala | 2 | 0.324 | 11 | 0.215 | 38 | 0.073 | 109 | 0.0147 |
|  | Eoacanthocephala | 2 | 0.324 | 3 | 0.059 | 9 | 0.017 | 35 | 0.0047 |
|  | Polyacanthocephala | 0 | 0.000 | 1 | 0.020 | 1 | 0.002 | 3 | 0.0004 |
|  | NA | 0 | 0.000 | 0 | 0.000 | 0 | 0.000 | 1 | 0.0001 |
| Rotifera | Monogononta | 3 | 0.486 | 21 | 0.410 | 49 | 0.094 | 718 | 0.0965 |
|  | Bdelloidea | 3 | 0.486 | 3 | 0.059 | 17 | 0.033 | 271 | 0.0364 |
|  | Seisonidea | 0 | 0.000 | 0 | 0.000 | 1 | 0.002 | 1 | 0.0001 |
|  | NA | 0 | 0.000 | 0 | 0.000 | 0 | 0.000 | 5 | 0.0007 |
| Nemertea | Enopla | 3 | 0.486 | 20 | 0.390 | 48 | 0.092 | 309 | 0.0415 |
|  | Pilidiophora | 1 | 0.162 | 8 | 0.156 | 33 | 0.063 | 221 | 0.0297 |
|  | Palaeonemertea | 0 | 0.000 | 4 | 0.078 | 6 | 0.011 | 112 | 0.0151 |
|  | NA | 0 | 0.000 | 0 | 0.000 | 1 | 0.002 | 61 | 0.0082 |
| Bryozoa | Gymnolaemata | 2 | 0.324 | 47 | 0.917 | 88 | 0.168 | 240 | 0.0323 |
|  | Stenolaemata | 2 | 0.324 | 9 | 0.176 | 14 | 0.027 | 23 | 0.0031 |
|  | Phylactolaemata | 0 | 0.000 | 5 | 0.098 | 8 | 0.015 | 16 | 0.0022 |
|  | NA | 0 | 0.000 | 0 | 0.000 | 0 | 0.000 | 3 | 0.0004 |
| Brachiopoda | Rhynchonellata | 3 | 0.486 | 12 | 0.234 | 22 | 0.042 | 40 | 0.0054 |
|  | Lingulata | 1 | 0.162 | 2 | 0.039 | 2 | 0.004 | 8 | 0.0011 |
|  | Craniata | 1 | 0.162 | 1 | 0.020 | 2 | 0.004 | 3 | 0.0004 |
|  | NA | 0 | 0.000 | 0 | 0.000 | 0 | 0.000 | 26 | 0.0035 |
| **Annelida** | **Polychaeta** | 16 | 2.593 | 84 | 1.640 | 593 | 1.135 | **4283** | **0.5759** |
|  | **Clitellata** | 12 | 1.945 | 43 | 0.839 | 304 | 0.582 | **3789** | **0.5095** |
|  | NA | 1 | 0.162 | 7 | 0.137 | 12 | 0.023 | 152 | 0.0204 |
|  | Phascolosomatidea | 1 | 0.162 | 0 | 0.000 | 0 | 0.000 | 0 | 0.0000 |
| Tardigrada | Eutardigrada | 3 | 0.486 | 5 | 0.098 | 27 | 0.052 | 213 | 0.0286 |
|  | Heterotardigrada | 2 | 0.324 | 3 | 0.059 | 15 | 0.029 | 104 | 0.0140 |
|  | NA | 0 | 0.000 | 0 | 0.000 | 0 | 0.000 | 2 | 0.0003 |
| **Nematoda** | **Chromadorea** | 10 | 1.621 | 116 | 2.264 | 414 | 0.792 | **2574** | **0.3461** |
|  | Enoplea | 9 | 1.459 | 32 | 0.625 | 85 | 0.163 | 660 | 0.0887 |
|  | NA | 0 | 0.000 | 0 | 0.000 | 0 | 0.000 | 13 | 0.0017 |
| Hemichordata | Pterobranchia | 2 | 0.324 | 2 | 0.039 | 2 | 0.004 | 2 | 0.0003 |
|  | Enteropneusta | 0 | 0.000 | 3 | 0.059 | 6 | 0.011 | 13 | 0.0017 |
|  | NA | 0 | 0.000 | 0 | 0.000 | 0 | 0.000 | 15 | 0.0020 |
| Onychophora | Udeonychophora | 1 | 0.162 | 2 | 0.039 | 33 | 0.063 | 198 | 0.0266 |
|  | NA | 0 | 0.000 | 0 | 0.000 | 0 | 0.000 | 1 | 0.0001 |
| Nematomorpha | Gordioida | 2 | 0.324 | 3 | 0.059 | 8 | 0.015 | 86 | 0.0116 |
|  | NA | 0 | 0.000 | 0 | 0.000 | 0 | 0.000 | 1 | 0.0001 |
| NA | Micrognathozoa | 1 | 0.162 | 1 | 0.020 | 1 | 0.002 | 1 | 0.0001 |
|  | NA | 0 | 0.000 | 0 | 0.000 | 0 | 0.000 | 6 | 0.0008 |
| Kinorhyncha | Cyclorhagida | 0 | 0.000 | 5 | 0.098 | 5 | 0.010 | 24 | 0.0032 |
|  | Allomalorhagida | 0 | 0.000 | 3 | 0.059 | 3 | 0.006 | 12 | 0.0016 |
| Ctenophora | Tentaculata | 4 | 0.648 | 4 | 0.078 | 5 | 0.010 | 20 | 0.0027 |
|  | Nuda | 1 | 0.162 | 1 | 0.020 | 1 | 0.002 | 6 | 0.0008 |
| Chaetognatha | Sagittoidea | 3 | 0.486 | 6 | 0.117 | 15 | 0.029 | 95 | 0.0128 |
|  | NA | 0 | 0.000 | 0 | 0.000 | 0 | 0.000 | 2 | 0.0003 |
| Xenacoelomorpha | NA | 2 | 0.324 | 20 | 0.390 | 48 | 0.092 | 175 | 0.0235 |
| Sipuncula | NA | 0 | 0.000 | 0 | 0.000 | 0 | 0.000 | 0 | 0.0000 |
| Priapulida | Priapulimorpha | 1 | 0.162 | 2 | 0.039 | 4 | 0.008 | 4 | 0.0005 |
| Placozoa | NA | 0 | 0.000 | 1 | 0.020 | 1 | 0.002 | 6 | 0.0008 |
| Phoronida | NA | 0 | 0.000 | 0 | 0.000 | 2 | 0.004 | 23 | 0.0031 |
| Gnathostomulida | NA | 2 | 0.324 | 6 | 0.117 | 8 | 0.015 | 10 | 0.0013 |
| Gastrotricha | NA | 2 | 0.324 | 11 | 0.215 | 33 | 0.063 | 124 | 0.0167 |
| Entoprocta | NA | 0 | 0.000 | 3 | 0.059 | 7 | 0.013 | 23 | 0.0031 |
| Dicyemida | NA | 0 | 0.000 | 1 | 0.020 | 2 | 0.004 | 16 | 0.0022 |
| Cycliophora | NA | 0 | 0.000 | 0 | 0.000 | 1 | 0.002 | 5 | 0.0007 |
